# Supplementary material for: The interactome of intact mitochondria by cross-linking mass spectrometry provides evidence for coexisting respiratory supercomplexes
Source: Mol Cell Proteomics. 2017 Dec 8;17(2):216–32. doi: 10.1074/mcp.RA117.000470 (PMC5795388; doi:10.1074/mcp.RA117.000470)
Supplement: Supplemental Data [file supp_RA117.000470_133922_0_supp_23978_fzffwf.zip › spectra_annotation/mito_DR_spectra_annotation/411-1-1-1-7-1.pdf]

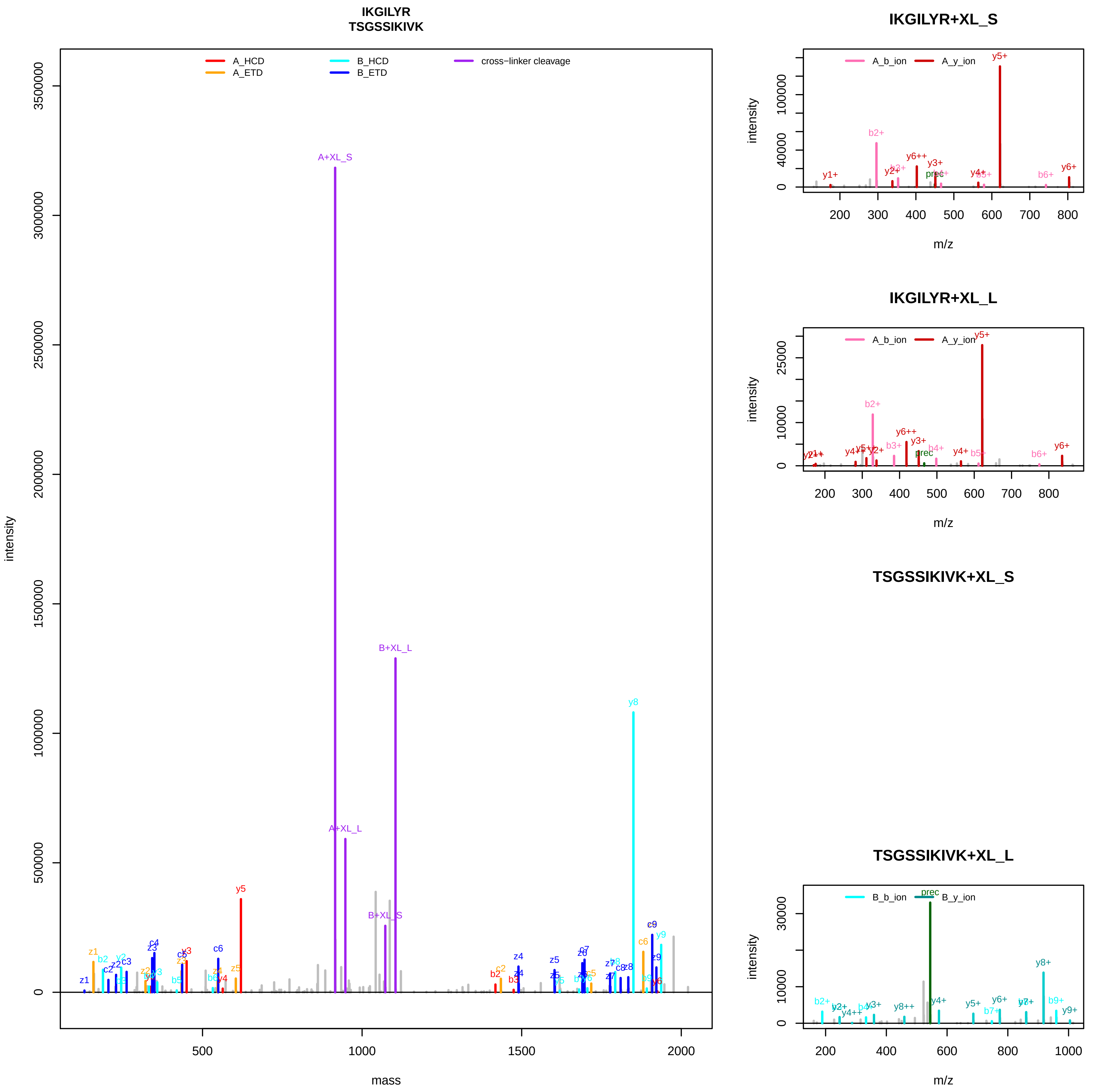

IKGILYR
TSGSSIKIVK
A_HCD
A_ETD
B_HCD
B_ETD
cross−linker cleavage
A+XL_S
B+XL_L
A+XL_L
B+XL_S
y8
y5
y9
c9
intensity
mass
0
500000
1000000
1500000
2000000
2500000
3000000
3500000
500
1000
1500
2000
IKGILYR+XL_S
A_b_ion
A_y_ion
y5+
b2+
y6++
y3+
prec
y1+
y2+
b3+
y4+
b5+
b6+
y6+
intensity
m/z
0
40000
100000
200
300
400
500
600
700
800
IKGILYR+XL_L
A_b_ion
A_y_ion
y5+
b2+
y6++
y3+
prec
b3+
b4+
y4+
b5+
b6+
y6+
intensity
m/z
0
10000
25000
200
300
400
500
600
700
800
TSGSSIKIVK+XL_S
TSGSSIKIVK+XL_L
B_b_ion
B_y_ion
prec
y8+
b2+
y2+
y4++
y3+
y8++
y4+
y5+
y6+
b7+
y7+
b9+
y9+
intensity
m/z
0
10000
30000
200
400
600
800
1000
